# Vital Statistics

**Published:** 1837-04-01

**Authors:** 


### VITAL STATISTICS.

1. A Statistical Account of the British Empire; exhibiting its Extent, Physical Capacities, Population, Industry, and Civil and Religious Institutions. By *J. R. M'Culloch*, Esq. assisted by numerous Contributors. In 2 Volumes 8vo, pp. 630-692. Charles Knight, London, 1837.

2. Medical Statistics, or the Causes and Effects of the Extension of Human Life. By *J. F. Palmer*, Senior Surgeon to the St. George's and St. James's Dispensary, &c. [From the Dublin Review.]

A statistical account of the British Empire was much wanted. Englishmen have long hugged themselves in the belief, that they excel all foreigners in occupations and in inquiries which practically affect the business of life. The long wars with the French, and our exclusion from the Continent, while they threw us on our national resources, encouraged our national industry, and exhibited our national energy, fostered at the same time that exclusive spirit, which has been said always to characterise islanders, and which, like individual conceit, is too apt to engender ignorance and repress improvement. It is now admitted that, in many respects, the Continental Nations excel us, and in few more than in the application of science to matters of administration.

This is not the place for any but medical discussions, and we have no intention of adverting to those parts of Mr. M'Culloch's elaborate and valuable work, which do not immediately affect us. In short, our business is with vital statistics, and vital statistics only.

A chapter is devoted to them in the second volume, and its composition has been entrusted to Mr. Farr, who appears to have executed it with ability and care. The chapter in question occupies thirty-five pages, and is too much condensed, and too circumstantial, to enable us to present a complete account of it. Indeed that would be unnecessary, as some of it is familiar to the medical reader. Another circumstance which precludes our pursuing the writer in detail, is his occasional disregard for simple and obvious arrangement.

The subjects to which we shall first advert, are those involved in the mortality of the population.

It appears that the annual mortality is about 2·13 per cent. That mortality varies with age, sex, occupation, and locality.

1. *Influence of Age on Mortality.* From birth to the age of puberty, the sickness and mortality decline; from puberty they increase slowly, but in a geometrical progression, up to the 50th or 60th year, and then more rapidly to the end. The following table will shew the ratio of mortality to age in the countries specified.

I. TABLE showing, in each of Thirteen Intervals of Age, the Number of Deaths which occur annually for every 1,000 Persons, living at the same Age, in England, Sweden, and Belgium.

| Between Ages. | England and Wales. | | | | | Carlisle. | Belgium. | Sweden. | |
|---|---|---|---|---|---|---|---|---|---|
| | Females, 7 Years, 1818-24. | | | 18 years, 1813-30. | | 9 Years, 1779-87. | 1829, both Sexes. | 21 Years, 1755-75, both Sexes. | 20 Years, 1776-95, both Sexes. |
| | Living in 1821. | Dying in 7 Years. | Annual Deaths per 1,000. | Males. | Females. | | | | |
| 0 and 5 | 774,689 | 239,482 | 45·6 | 53·5 | 46·0 | 82·3 | 65·8 | 90·1 | 85·0 |
| 5 10 | 682,457 | 30,173 | 6·5 | 7·2 | 6·7 | 10·2 | 8·7 | 14·2 | 13·6 |
| 10 15 | 569,366 | 20,244 | 5·3 | 5·0 | 5·2 | 5·0 | 5·4 | 6·6 | 6·2 |
| 15 20 | 535,569 | 27,391 | 7·6 | 7·2 | 7·6 | 6·8 | 6·6 | 7·6 | 7·0 |
| 20 30 | 901,338 | 61,701 | 10·1 | 10·1 | 10·4 | 7·5 | 9·1 | 9·2 | 8·9 |
| 30 40 | 649,507 | 53,417 | 12·1 | 11·4 | 12·4 | 10·6 | 10·0 | 12·2 | 11·6 |
| 40 50 | 500,977 | 50,214 | 14·8 | 14·9 | 14·9 | 14·3 | 13·6 | 17·4 | 16·1 |
| 50 60 | 352,160 | 49,671 | 20·8 | 23·4 | 21·6 | 18·3 | 21·7 | 26·4 | 23·9 |
| 60 70 | 249,184 | 67,918 | 40·2 | 45·3 | 41·2 | 41·2 | 38·5 | 48·1 | 49·3 |
| 70 80 | 124,648 | 80,138 | 94·9 | 101·2 | 96·9 | 83·0 | 90·9 | 102·3 | 104·1 |
| 80 90 | 36,315 | 52,318 | 212·7 | 227·1 | 214·6 | 175·6 | 178·8 | 207·8 | 197·4 |
| 90 100 | 3,280 | 8,169 | 367·8 | 370·1 | 371·9 | 284·4 | 304·7 | 394·1 | 351·3 |
| Above 100 | 129 | 512 | 586·1 | 611·1 | 560·6 | | | | |
| Ages specified .. | 5,379,619 | 741,348 | 20·3 | 21·7 | 20·7 | 25·0 | 22·7 | 28·9 | 26·8 |
| Ages omitted .. | 765,090 | 27,157 | | | | | | | |
| Total enumerated | 6,144,709 | 768,505 | | | | | | | |
| Estimated omission ..... | | 106,780 | | | | | | | |

It would seem that the mortality of the whole English population, between the ages of 20 and 40, was higher than in Belgium and Sweden. The mortality of children, under five years of age, has materially decreased in England within the last century—a fact à priori probable, and not at all inexplicable. In London, the bills of mortality prove that, in the 20 years, 1730-49, out of 100 born, 74·5 died under the age of 5 years; while, in the 20 years, 1810-29, only 31·8 died out of the same number.

2. *Influence of Sex on Mortality.* Professors of statistics would seem to commit blunders like less exact folks, or, at all events, to disagree to a considerable extent. Nay, there is among them not a little of that spirit which distinguishes carpenters, tailors, members of trades, two of whom are said to be never capable of a very lively friendship. Thus, Mr. Edmonds abuses Mr. Rickman and Mr. Finlaison, and Mr. Farr would appear to cleave to Mr. Edmonds. But this by the way.

If any point was considered established it was the superiority of female over male life. Nay, a Life Assurance Company in London has, within these few months, acted publicly on that conclusion, and lowered the premium on female insurers. But this would appear to be a delusion, and the Assurance Company would seem likely to make a sorry bargain.*

Contrary, says Mr. Farr, to the Swedish observations, the mortality of females, between the ages of 10 and 40, is higher than that of males; it is only in childhood and after the 50th year, that the mortality of females is lower than that of males.

3. *Differences of Mortality in the Counties, Towns, &c. of England.* Some of the counties of England are much less healthy than others are. Cornwall and Devon are the healthiest—Cambridge, Kent, Surrey, and Middlesex the reverse. The counties in which the greatest number of inhabitants are congregated in towns, are generally the most unhealthy.

Mr. Edmonds, whom Mr. Farr copies, divides the English counties into eight classes. *In class* 1, are Cornwall and Devon.—*In class* 2, Wales and Monmouth.—*In class* 3, Dorset, Somerset, Wilts, Gloucester, Hereford, Northumberland, and Cumberland.—*In class* 4, Westmoreland, North York, Rutland, Norfolk, Suffolk, and Hertford. *In class* 5, Durham, East York, West York, Leicester, and Lincoln.—*In class* 6, Salop, Derby, Northampton, Huntingdon, Essex, Bedford, Bucks, Oxford, Berks, Southampton, Sussex. *In class* 7, Lancaster, Chester, Nottingham, Stafford, Warwick, Worcester, Cambridge, and Kent. *In class* 8, Surrey and Middlesex.

The mortality of the inhabitants of the counties, thus arranged, is displayed by Mr. Edmonds in the following table.

---

* We can state as a fact, that a large and ably-conducted Life Assurance Company in London, has met with its greatest losses on its female lives. It has had no inducement to lower the rate of insurance on those lives. On the contrary, it would willingly reject all insurances on women; and, but for the loss of business and the odium which such a singularity might entail on it, it is far from impossible that such a step would be taken. It is probable that many circumstances contribute to diminish the value of *insured* female lives. The preliminary examinations of a woman's state of health can seldom be so searching, or at least so accurate, as those of a man's. Women do not scruple to deceive, and a practical acquaintance with their habits and their conduct establishes the conviction that, in *all* classes, they possess a feebler sense of the obligation of truth, in the ordinary business of life, than men have. Why this should be, we leave to casuists to determine. Assurance Companies have discovered that it is so.

TABLE showing, in each of Six Gradations of Age, the Mortality per cent. of each Sex in each of Eight Classes of English Counties.

| Class. | Males (corrected).* | | | | | | | Females. | | | | | | |
|---|---|---|---|---|---|---|---|---|---|---|---|---|---|---|
| | 0·5 | 5·10 | 10·15 | 15·30 | 30·60 | Above 60 | All Ages. | 0.5 | 5·10 | 10·15 | 15·30 | 30·60 | Above 60. | All Ages. |
| | | | | 1 | 10 | | | | | | | | | |
| 1 | 4·19 | ·63 | ·38 | ·75 | 1·36 | 7·58 | 1·90 | 3·65 | ·63 | ·41 | ·70 | 1·18 | 7·14 | 1·78 |
| 2 | 3·87 | ·60 | ·44 | ·83 | 1·31 | 7·46 | 1·83 | 3·39 | ·59 | ·43 | ·75 | 1·26 | 7·06 | 1·79 |
| 3 | 4·14 | ·63 | ·44 | ·76 | 1·32 | 7·15 | 1·86 | 3·58 | ·59 | ·47 | ·83 | 1·35 | 6·96 | 1·83 |
| 4 | 4·36 | ·58 | ·44 | ·80 | 1·23 | 7·30 | 1·92 | 3·76 | ·55 | ·52 | ·92 | 1·35 | 7·12 | 1·90 |
| 5 | 5·24 | ·72 | ·52 | ·86 | 1·38 | 7·47 | 2·09 | 4·52 | ·64 | ·52 | ·92 | 1·47 | 7·33 | 2·00 |
| 6 | 4·45 | ·62 | ·47 | ·87 | 1·40 | 7·62 | 1·99 | 3·81 | ·58 | ·55 | 1·05 | 1·50 | 7·51 | 1·96 |
| 7 | 6·11 | ·78 | ·56 | 1·03 | 1·64 | 7·64 | 2·34 | 5·33 | ·71 | ·56 | 1·03 | 1·65 | 7·48 | 2·18 |
| 8 | 8·19 | 1·05 | ·53 | ·95 | 2·27 | 10·72 | 2·88 | 6·68 | ·95 | ·53 | ·85 | 1·91 | 9·95 | 2·50 |
| | | | | 1· | 93 | | | | | | | | | |
| Totals | 5·30 | ·72 | ·49 | ·88 | 1·53 | 7·77 | 2·15 | 4·56 | ·66 | ·52 | ·93 | 1·52 | 7·53 | 2·05 |
| | | | | 1· | 33 | | | | | | | | | |
| | Table of "Mean Mortality," when period of "Infancy" terminates at seven years. | | | | | | | 4·47 | ·77 | ·65 | ·86 | 1·66 | 7·62 | 2·09 |

* The apparent mortality of the male sex between 15 and 30, between 30 and 60, and at all ages, has been diminished by ·06 in the total, and in classes 1, 2, and 3; it has been diminished by ·03 in classes 4, 5, 6, and 7; and by ·10 in the eighth class.

The mortality of cities is highly interesting, not only to their inhabitants, but to the whole nation. The grandeur of a people depends on *their* multiplication, their wealth, and populousness. If the marts of commerce are, as Dr. Price has designated them, the "graves of men," we must first deplore—then attempt to remedy the evil. A necessary evil, to some extent, it probably must ever be; yet the history of London is sufficient to prove that much has been done, and to render it probable that more may be done still, to augment the salubrity of cities.

A table is given for the purpose of shewing the increasing mortality in Glasgow. It shews, too, the lesser mortality of females in that city—a fact treated by Mr. Farr as a gross blunder on the part of Mr. Finlaison. It appears from the table, that between the ages of 30 and 60, the mortality of each sex has increased 20 per cent. every five years between 1821 and 1835. This is attributed in part to the augmented influx of the Irish, who introduce their national habits and national fevers. By a report of the Glasgow Infirmary, it seems that, in 1835, out of 3,260 patients treated, 1,258 had fevers, and of these 125 died.

The relative mortality of England, Scotland, and Ireland cannot at present be determined. But the following calculation may lead to an approximation to the truth. The number, above the age of 60 years, alive, for every 100 living between the ages of 30 and 60, was, in—

| | | |
|---|---|---|
| England and Wales.. | (Females) .......... | 27·5 |
| Scotland.......... | (Females) .......... | 28·0 |
| Ireland ...... | (Males and Females) .... . | 15·7 |
| Belgium...... | (Males and Females) ...... | 30·3 |

4. *Influence of Foreign Stations on our Army.* This is shewn in a table compiled by the diligence of Mr. Marshall, a highly intelligent surgeon.

TABLE of the Mortality of the British Army, showing the mean Number of Annual Deaths out of 100 living at each Station mentioned.

| Time and Place of Observation. | English Army. | Extent of Observations. | | Annual Rate of Mortality per Cent. | | |
|---|---|---|---|---|---|---|
| | | Average Force. | Years. | Maximum. | Mean. | Minimum. |
| *The United Kingdom .. | British Army...... | 46,460 | 10 | ...... | 1·5 | |
| *Ireland, 1797-1828.... | Ditto .......... | 36,921 | 32 | 2·0 | 1·5 | 1·1 |
| *Mediterranean.* | | | | | | |
| *Malta, 1824-31 ....... | The garrison ...... | 2,226 | 8 | 2·8 | 1·5 | 1·0 |
| *Gibraltar ............ | Ditto .......... | 3,267 | 17 | 13·4 | 2·0 | 0·7 |
| *Ionian Islands........ | The troops........ | 3,467 | 13 | 3·6 | 2·6 | 1·4 |
| *East Indies.* | | | | | | |
| *Fort St. George Presid. } *Madras, 1827-30.... } | (1) European troops | 11,820 | 4 | 7·1 | 4·8 | 3·2 |
| | Native troops ..... | 69,550 | 4 | 1·6 | 1·4 | 1·0 |
| *Bengal, 1826-32 ...... | (2) European troops | 8,700 | 7 | 9·7 | 5·7 | 3·8 |
| *West Indies.* | | | | | | |
| *Windward Islands . } | 1796-1805, ditto .. | 13,610 | 10 | 27·7 | 18·3 | 8·0 |
| *Leeward Islands .... } | 1810-28, ditto .... | 5,768 | 19 | 23·4 | 11·3 | 4·7 |
| *Jamaica Honduras, 1810-28. ...... } | Ditto . ........ | 2,528 | 19 | 47·2 | 15·5 | 7·8 |
| *Jamaica, Honduras, Windward and Leeward Islands ...... } | Colonial troops, (Blacks)...... } | 2,733 | 19 | 8·4 | 5·5 | 1·8 |

The mortality of the native troops in the Madras Presidency was only 1·4 per cent.; that of the European troops was quadrupled in Bengal. At home 1·5, in Bengal 5·7 per cent. of the European troops died. The climate of the East Indies is, therefore, congenial to the Hindoos, and only extraordinarily fatal to foreigners.

The mortality of the French infantry is greater than that of our own army on the home station. Its mean annual rate per cent. is, for the troops of the line, 2·00—for the Garde Royale, 1·47—for the whole army, 1·94.

Mortality is usually the result of sickness. The former has been reduced by the formulæ of calculation to something approaching to certainty and rule. Will sickness submit to similar calculations? At first sight it might appear difficult, if not impossible, to procure adequate data; nay, à priori, it does not *seem* to follow, that because death observes an appreciable ratio to age and sex, sickness will do so too. It is certain that the laws of sickness can never be determined with any thing like the same precision as are those of death—because death is chronicled in all classes, and sickness is not. It is only under peculiar circumstances, that attacks of sickness are regularly, or may be regularly registered—amongst official employés, whose absence is felt, and whose illness must be certified. This is one obstacle to accuracy. But the very term sickness is indefinite. One person will lie upon the sofa for a cold—the mechanic, affected with the same complaint, will pursue his unceasing and laborious occupation. Some persons, of weak habits and infirm health, may be said to be always labouring under sickness—that is, they suffer ailments which would utterly disable from active employments, but which yet suffer them to engage in sedentary or light occupations. A diligent pursuit of the details of sickness—a careful observation of the habits of individuals and of classes, would enable the inquirer to ascertain and to point out the numerous sources of fallacy and of doubt, that must surround the attempt to enunciate a law of sickness.

The benefit societies, whose necessities have forced, and whose circumstances have enabled them to calculate, to some extent, the liabilities to sickness, arrange *that* under three heads:—*Bedfast* sickness, when the individual is absolutely incapable of exertion—*walking* sickness, when the patient is incapable of entire attention to his concerns, as he is in many chronic diseases, after reduced dislocations, and so forth—and *permanent* sickness, as is the case of the paralytic, the maimed, the incurables.

From a table, shewing the proportion of sick out of 100 living at each interval of age, in friendly societies, it appears that, between the ages of 30 and 40, 1·32 are constantly sick in the Scotch benefit societies, and 1·83 in the English. Between the ages of 60 and 70, 10·80 are ill in the former, and 11·26 in the latter. Thus, the sickness increases with the age. The time for all ages is 2·45 in the Scotch societies—2·76 in the English.

In the parish of Methven, in Perthshire, it was ascertained that 35 out of 743, or 4·7 per cent. of the male population, above 15, would not, from bodily or mental infirmity, have been admitted as members of the friendly societies at all.

Bad health, however, and sickness are not, in the poorer classes, equivalent terms. Thus, Dr. Forbes ascertained, by personal examination of 120 Cornish miners, in actual employment, that only 63 had good health; of the remaining half, 26 had difficulty of breathing, 14 pain of chest, 10

pain of stomach and bowels, 5 lumbago, pain of shoulder, palpitation, scrofula, or fits. Out of 115 children below 18 years of age, Dr. Bisset Hawkins states, that 84 had good health; 25 middling health; 6 bad health. Of the miners at work, only 53—of the factory children only 73, per cent. enjoyed good health.

A return has been made from the various dock-yards, of the numbers of workmen sick in the years 1830, 1831, and 1832, and the following table exhibits a condensed view of the facts that they convey.

TABLE presenting a condensed View of the above Facts.

| Years. | Average Number of Men. | Number of Cases. | | Days of Sickness from spontaneous Disease. | Days of Sickness from Injuries. | Total Days of Sickness. |
|---|---|---|---|---|---|---|
| | | Diseases. | Hurts. | | | |
| 1830 | 2,079 | 697 | 357 | 9,188 | 5,884 | 15,072 |
| 1831 | 2,002 | 888 | 325 | 9,605 | 4,620 | 14,225 |
| 1832 | 1,867 | 665 | 329 | 8,617 | 5,086 | 13,703 |
| 3 years | 5,939 | 2,250 | 1,011 | 27,410 | 15,590 | 43,000 |

This table furnishes, as the mean of the three years, the following interesting results. In the year, 1 man in 6 is seriously hurt; 2 in 5 fall ill. Each man, on an average, has an attack of illness, either spontaneous or caused by external injury, every 2 years; and, at an average, each disease lasts 14 days. Mr. Farr goes on to observe, that it may safely be assumed that, of the 21,000 labourers employed in the dock-yards, 2 per cent., or more than 420, are constantly kept at home by diseases, two-thirds of which are independent of accidents.

The most accurate report which has been yet published would seem to be that of the state of health among the men, employed by the East India Company in London. It is contained in a supplementary report, by Dr. Mitchell, to that of the Factory Commissioners. It was "in the form of a large volume, containing a list of 2,461 labourers, employed in the month of April, 1823, with a statement of the number of days' illness experienced by these labourers, one by one, year by year, for the ten succeeding years; also the date of every death, and the date when any labourer ceased to be employed, by being superannuated and pensioned, dismissed, or by voluntarily leaving the service of the Company."

Every labourer put upon the sick list is allowed 1s. 6d. a day, Sundays included; he is also seen every day by the surgeon, and, therefore, remains no longer absent than the case requires.

During the 10 years, 496 died, 248 were pensioned, and 208 left the service or were dismissed. The reporter, Dr. Mitchell, has calculated a table of the duration of sickness per annum for every age, from 16 to 81, which we subjoin:—

| Age. | Average Duration of Sickness per Ann. for every Man employed. | Average Duration of Sickness for every Man sick. | Age. | Average Duration of Sickness per Ann. for every Man employed. | Average Duration of Sickness for every Man sick. |
|---|---|---|---|---|---|
| | *Days.* | *Days.* | | *Days.* | *Days.* |
| Under 21 | 4·02 | 13·96 | 51 to 61 | 7·00 | 28·60 |
| 21 to 31 | 4·94 | 18·70 | 61 — 71 | 10·08 | 29·07 |
| 31 — 41 | 5·06 | 22·63 | 71 — 81 | 11·63 | 31·77 |
| 41 — 51 | 5·31 | 23·21 | | | |

From other calculations and another table, it appears that the annual rate of mortality was 3·13 per cent. Notwithstanding the selection of healthy men as labourers, the deaths between the ages of 40 and 60, agree very nearly with the general mortality of males in London, between the years 1813 and 1830. Amongst the labourers, from 40 to 50, the annual mortality was 2·43 : amongst the male inhabitants of London, it was 2·54— amongst the males in Stockholm, from 1755 to 1763, it was 4·67. Between the ages of 50 and 60, the mortality of the labourers was 4·27—of males in London, 4·04—of males in Stockholm, 6·46.

The mortality under 40, observes Mr. Farr, is not so high among the labourers, because the greater part of them are selected healthy men, received into the service between the age of 20 and 35; after 50 it is higher than the general mortality in London. These men were well supplied with food and clothing; their work without being hard insured regular muscular exercise; in sickness they had rest and proper medical attendance; yet between 40 and 50 the mortality was 67 per cent., between 50 and 60 as much as 82 per cent. higher than the mortality at the same ages in all England. Such facts as these annihilate the supposition, that the increased mortality in cities is due to want of food and greater misery; nor, although these men drank freely, can we admit that their moral habits differed so greatly from those of country labourers as to account for their greater mortality.

Of the 2,461 labourers, 10 per cent. were pensioned in the course of ten years; 6 per cent. were discharged, or quitted the service; 1 man in 81 working a year was pensioned; 1 in 4 had an attack of sickness; 1 in 60 was constantly on the sick list; 1 in 21 (4·79 per cent.) of the labourers was a pensioner; and 1 in 6 of the pensioners died annually. Some other observations on this head we pass over. They merit the attention of benefit societies, but do not require our's.

Two tables are given of the sickness of factory labourers in Lancashire and Glasgow. But it seems that they are open to objection, and we therefore pass them by. So far as they go, they exhibit a greater amount of sickness in the females than in the males, both in Lancashire and Glasgow.

The mortality of the English army is higher than the mortality of the general population; and the sickness, including every class of disease, is more than twice as great as that recorded in the preceding tables, at the ages 20-35, which correspond nearly with the ages of the troops. The mean proportion sick per cent. in Ireland (1797-1828), observed on an average force of 36,921 men, amounted to 5·1 ; but a certain proportion of the sickness in the army is from syphilis, and this is not included in the other observations. In the English army—cavalry, foot-guards, and infantry—Mr. Finlaison has estimated the numbers of sick, in the years 1823-4, as somewhat more than 4 per cent.

The sick time in Madras, remarks Mr. Farr, in 1808-9 was 12·4 per cent. of the lifetime. Annesley considered 10 per cent. in that climate unhealthy ; and this appears to be near the mean proportion of European troops constantly sick in the East Indies. In places where the mortality is high, the rate of sickness fluctuates very much from year to year ; and, exclusive of losses in battle, the mortality and sickness are tripled or quadrupled in a campaign. This kind of knowledge is still imperfect, although indispensable to those who would employ masses of men with effect in different circumstances.

5. *Morbility of the Population.* Passing over some tables, we introduce a rather lengthened extract, which contains the results of many different inquiries.

"The attacks of disease vary in frequency to a great extent in unhealthy and salubrious situations: but the experience of the East India Company's labourers, of the children belonging to the Bennet-street School, which has the best-regulated sick society of any in Manchester, and of the artisans of the Trades Club in Wurzburg, all receiving pay during sickness, and only falling on the funds in cases of some duration and severity, tends to shew that 100 of the efficient male population of this country are not liable to more than 25 severe attacks of disease in the year. Each man is liable to a protracted disease, disabling him from work, every four years: this forms one great section of the sickness of the country ; but it does not include syphilis, accidents from fighting and drunkenness, or the many ailments which make men apply for medical advice, while they carry on their occupation, comprising, perhaps, as many more cases of a slighter character, which raise to 50 per cent. the proportion of the population attacked annually. In the Portsmouth dockyard 37·8 per cent of the men fall ill, 16 per cent. meet with accidents in the year : besides accidents, the attacks of sickness in Sheerness amounted to 43·7 per cent. The reported attacks of sickness in Sheerness are exclusive of accidents; and if, as is probable, the same exclusion was made in the other returns, the spontaneous cases amounted to nearly 50 per cent. in Chatham and Pembroke. By excluding the slighter cases the attacks of sickness may be reduced near the level of the preceding series : in the healthiest dockyard, Plymouth for example, out of 2,147 cases, 635 did not exceed three days' duration ; and by subtracting these, the proportion attacked in 100 will be reduced from 34·7 to 24·4. Accidents, although excepted here, ought not to be excluded, because they occur as common inevitable causes of disease among all classes of the people, and raise the sickness to a considerable, although varying, extent ; in Portsmouth, the increase of cases from this cause was 42 per cent. Except Plymouth, the dockyards appear to fall somewhat below the national standard of health ; more than 50 in 100 of the men are attacked by sickness of one sort or other annually. In Sheerness, out of 1,422 cases of sickness, 263 were agues, 142 rheumatisms, 68 colics and cholera. The Report of the Liverpool Society, by the surgeon, Mr. Parr, shews that 57·2 per cent. of the members receive medical advice in the year. Mr. Parr makes a useful distinction between the patients attended at their own houses and those seen at his surgery : the first, not quite comprehending all disabled for work, were

17·8 per cent., nearly a third of all the patients treated. Among 100 members, 8·6 met with accidents in the year. The return from the Deanston Cotton Works, in Scotland, after deducting cases of less than three days' duration, makes the annual attacks of sickness among males 20, among females 44·8 per cent. This difference between males and females deserves attention; it is produced by rheumatisms, diarrhœas, and even wounds, but more particularly by catarrh and headach. The female cases approach the nearest to the total attacks; as the wages of women are lower, and they give over working abroad on slighter occasions than males. Almost an equal difference is visible in the Lancashire cotton works; in Glasgow the difference is trifling. Females leave off work, and apply for medical advice, more frequently than males; but as the mortality between 15 and 40 is but a little higher, the same must hold respecting severe illnesses. The cases observed in factories, only reported on the recollection of the workmen, would very likely include nearly all the serious diseases, and a varying proportion of the slighter distempers which detained them from work. The attacks of sickness among paupers—the feeble, crippled, maimed, idiotic, crazy, miserable, dirty, dissolute, vicious, unfortunate—rejected as refuse from all the foregoing classes, are more numerous than the attacks to which select labourers are liable; and medical men, in undertaking to attend paupers, should bear this in recollection. For the facts relating to English prisoners we are indebted to a highly interesting paper of Mr. Chadwick, in which, by collating the dietaries of 60 prisons in England and Wales, he has rendered it probable that the lowest prison *diet* does not increase sickness. An accurate return of the average population of the prisons, the ages of the prisoners, the diseases and deaths, the amount of labour performed, and information concerning the ventilation of the cells, will, in a few years, settle this question: the average population in this table was assumed to be furnished by the numbers remaining at five times in each of the 60 prisons.

We are unacquainted with any data from which the absolute morbility of the British population can be deduced, any more than the mean duration of each case and the mortality of the sick, with which it is intimately connected. Several interesting observations on distinct classes, and on the morbility and duration of sickness at different ages, however exist. At the age of 20-50, the duration of each attack among the East India Company's labourers was 22·2 days, and with the pension time 36 days: 7·8 in 100 cases died, or 7·4 exclusive of pensioners. The duration of these cases approaches very near the term of treatment in 14 Paris hospitals, where patients are admitted indiscriminately and continue till they recover or die; in 1819-1825, the mean number, 3,947 patients, remained 35 days. In the hospitals of this country, where adults and more chronic cases are received, but at the same time are often sent out before death occurs, the patients continue longer under treatment; in the county hospitals (31) of Ireland, 38·3 days; of England, 39·4 days; of the metropolis, 42 days. Where slighter and ephemeral cases are counted, and organic diseases are excluded, the duration of cases among adults does not, according to the returns from the dockyards, exceed 12 days. In Corfu, 1816-1821, the mean duration of all the diseases (14,098) was 16·1 days.

It is of great importance for medical men to know the average number of deaths in all the cases that come under their care, in order to judge of the remedial influence of medical appliances. The hospitals furnish some, although inadequate information on this head. The sanability of the sick appears to decrease in the large cities; partly, it may be, because more serious accidents are admitted." 583.

| Hospitals. | In-Patients treated in 1 Year. | Mean Number of In-Patients. | Deaths. | Deaths out of 100 Patients. | Deaths in 36·5 Days out of 100 Patients. |
|---|---|---|---|---|---|
| Salop, 4 years (1830—33).... | 955 | 92 | 34 | 3·7 | 3·6 |
| Winchester, 1 year (1833—34) | 798 | 83 | 31 | 3·8 | 3·7 |
| Salisbury, 1 year (1833—4) .. | 894 | 88 | 27 | 3·1 | 3·1 |
| Chester, 2 years (1833—5) ... | 489 | 45 | 20 | 4·2 | 4·5 |
| Manchester, 1 year (1831—2) | 1,784 | 128 | 128 | 7·2 | 10·0 |
| Liverpool, 1 year (1831) ..... | 1,960 | 220 | 109 | 5·6 | 5·0 |
| Bristol, 1 year (1828—9) .... | 1,483 | 195 | 160 | 9·5 | 8·2 |
| London, 7 hospitals.... ..... | 18,740 | 2,191 | 1,605 | 9·0 | 7·6 |

Men placed under the same circumstances, appear equally liable to an attack of sickness between 11 and 60 years of age; 100 of the London labourers, in each of the decennial periods between 20 and 60, had nearly 23·5 attacks of sickness annually.

Two other positions are laid down:—1, That the mean duration of each case increases as age advances; 2, that the mortality among the attacked augments with age, at the same rate as the mortality among the entire number living.

"The sick time increases with age in a geometrical progression. If, therefore, the number of attacks at each age be the same, the duration of each attack will increase in the same ratio; and conversely, if the duration of the cases and the sick time augment at the same rate, the number of attacks at every age will be equal. Any two of the elements being given, the third may always be deduced from them. Again, if the mortality of the attacked increase at the same rate as the mortality of the entire population, the proportion attacked at every age will be the same. Among the London labourers, the mortality between 30-40, 40-50, was 1·48, 2·43 in 100 living; the mortality among 100 attacked was 6·5, 10·4. Now 1·48 is to 2·43 very nearly as 6·5 is to 10·4; and it results from this that the attacks, whatever their absolute number may be, whether 22 or 52, were the same in both periods." 585.

6. *Diseases.* There is not much in the observations upon these to give us pause. The writer enters into a calculation to shew that, if the same number of persons died by ordinary chronic diseases, who at times die from short and epidemic ones, a vast deal of misery and a great deal of inconvenience would ensue. As this may readily be granted, we pass on. Has insanity increased? The returns are yet unequal to the solution of this question, for an obvious source of fallacy sticks to them. Formerly, the gross ill-treatment of lunatics must have shortened their existence. Now they live longer, and, consequently, more will be alive at a given time, independently of an increase of numbers relatively to the population. The same fallacy applies to the present estimates, between the numbers of insane in town and country. The superior longevity of the latter may similarly and deceptively swell their numbers. Sir A. Halliday calculated that, in England, 1 in 1000 of the population is insane—in Wales, 1 in 800—and, in Scotland, 1 in 574. But a calculation, more exact in its data, of the mad amongst the Quakers, makes *them* amount to 3·4 in 1000—and perhaps this is an approach to what obtains among the more opulent classes in England.

In the paper of Mr. Palmer, we find some further observations on insanity, which we may here introduce. The points to which we shall advert are, the diminished mortality of lunatic asylums, and the comparative frequency of the disease in men and women.

It appears (says Mr. P.) by nearly all the returns of Europe, excepting France, that men are more prone to insanity than females, in the proportion of 100 to 77 : but this apparent discrepancy may, perhaps, be referred to the delicate feelings of relatives, who object to the idea of placing the female branches of their families in public asylums, especially as they are more easily controlled at their own homes or in private families. The proportion of females cured at the Bethlem Hospital, during the last 15 years, has been 47·0 per cent.; and at St. Luke's, 44·8 per cent.; the respective numbers for males being 39·6 and 41·3.—Of 997 curable patients, admitted at the former, from 1830 to 1834 (inclusive), considerably more than half were between the ages of 20 and 40, or precisely at that period of life when the passions acquire their greatest ascendancy. The mortality, during the same period, was a little less than 1 in 25, which is surprisingly small, when we consider that many of these labour under actual organic disease of a vital part. Of the recoveries, nearly one-half took place within the first six months.

In connexion with insanity, we may not unnaturally glance at the suicide. Is he more frequently an Englishman than a foreigner? Popular credulity, Continental abhorrence of our customs and our manners, and our own paradoxical love and abuse of our country, our climate, and ourselves, have stamped self-destruction as a national characteristic, and as the peculiar offspring of the gloom and fogs of November. Exact investigations absolve us from this national weakness or crime, and prove that we are less suicidally inclined than the German or the Frenchman.

About 100 instances only of suicide annually occur in the metropolis. From 1812 to 1824, continues Mr. Palmer, the total number of suicides for the City of Westminster was only 290, or 1 in 8000—a proportion at least three times inferior to that for any of the great cities of France or Germany; and if allowance is made for the extreme dissipation of many parts of this city, we shall not be far wrong in considering 1 in 10,000 a medium proportion. In Prussia, the civic cases are to the rural as 14 to 4. The propensity is stronger in the male than in the female, as 5 to 2, in this country, and 2 to 1 in France. Many curious examples are recorded of the influence of imitation, in determining the thoughts to suicide, especially of those who are predisposed. The shocking recital of horrible cases in the newspapers, is attended with this effect. It has occasionally become necessary for the public authorities to interfere, and either to deny the rites of Christian burial, or to expose the corpse to some indignity, in order to arrest the progress of a suicidal contagion. Dr. Caspar relates the existence of a suicidal club in Prussia, consisting of six persons, all of whom accomplished their purpose ; and a similar club is said to have existed in Paris not long since, the members of which bound themselves by a regulation that, every year, one of their number should be selected to destroy himself as a testimony of their sincerity. Among the causes of suicide, sheer misery holds a prominent place ; next to that, domestic unhappiness ; then reverses of fortune, disappointed love, and gambling. A great number of attempted suicides arise from the loss of female honour, accompanied by the prospect of pregnancy. It is remarkable, that this disposition is far more predominant in Protestant than in Catholic communities. In Spain it is so extremely rare, that for the whole of 1826, only 16 instances were officially reported. From 1812 to 1824, the suicides committed in Westminster, in the months of June, were 34, and those in the months of November, 22. In 1812, 1815, 1820, and 1824, the months of November did not afford a single case.

We are next presented with tables, exhibiting the fatal diseases of London for the last two centuries. They are distributed in six columns, and embrace the following periods of time. Column A is deduced from 65,706 observations, made in the seven years, 1629-35. Col. B, from 426,253 diseases, recorded in the twenty years, 1660-79. Col. C from 732,873 deaths, occurring in the thirty years, 1728-57. Cols. D and E each embrace periods of ten years, 1771-80 and 1801-10. While col. F presents the results of the five last bills of mortality, 1831-5, and the actual fatal diseases of London, so far as they are shewn by the present system of registration. The table containing these six columns, exhibits the relative fatality of a vast number of existing, and of many forgotten diseases. Thus, the "chrisomes," "overlaid," "mold-shot head," "liver-grown," "griping of the guts," "rising of the lights and mother," "jaw-fallen," "planet-struck," raise their grisly and portentous fronts in the old bills, but, pressed by modern science and nomenclature, slink out of the new.

This table is too long for us; but there is another, equal in interest, and superior in conciseness, which displays the fatality of the principal diseases in the several periods we have mentioned. The observations are made on 100,000 living in each period, and the whole mortality, as well as the particular, are shewn.

[*See Table on the following page.*]

We observe, first, that the whole mortality has materially decreased. Fever, plague, dysentery, cholera, (with the exception of the late epidemic,) and small-pox, have diminished in a striking manner. Measles and scarlet-fever have increased, yet, as Mr. Farr observes, the mortality of all three diseases—of small-pox, measles, and scarlet fever conjoined, is only half as great as that formerly occasioned by small-pox alone. Fever has declined in nearly the same ratio as small-pox. Consumption has decreased. It would appear that fevers and consumption prevail together. It is very prevalent among the British troops in the West Indies. We know that the same causes tend to produce both fevers and consumption. Insufficient food and clothing, and exposure to wet and cold, are prolific sources of both.

7. *Fatal Diseases at different Ages.* The data for establishing this desirable information are not numerous. But the accurate registration which the working of the Act lately passed ensures, will speedily remedy the deficiency that we complain of. Seven tables, founded on the data that we have, are given in the work before us. But they are too long for us, and their insufficiency will justify our neglect of them. From the remarks appended to them, we shall make two extracts.

1. "What is the effect of the selection exercised by the assurance companies?

TABLE, showing the mean annual Number of Deaths in London produced by 20 Classes of Disease out of 100,000 living.

| By | A. 1629—35. | B. 1660—79. | C. 1728—57. | D. 1771—80. | E. 1801—10. | F. 1831—5. |
|---|---|---|---|---|---|---|
| Chrisomes, overlaid, convulsions, worms, teething, mold-shot head, dropsy on the head, inflammation of brain, rickets, livergrown, canker, thrush, croup, hooping-cough | 1,681 | 1,591 | 1,827 | 1,682 | 789 | 625 |
| Small-pox | 189 | 417 | 426 | 502 | 204 | 83 |
| Measles | 16 | 47 | 37 | 48 | 94 | 86 |
| Scarlet-fever | ... | ... | ... | ... | ... | 53 |
| Fever | 636 | 785 | 785 | 621 | 264 | 111 |
| spotted | 45 | 90 | | | | |
| Plague | 125 | 1,223 | | | | |
| Dysentery | 221 | 894 | 50 | 17 | 1 | 1 |
| Surfeit or cholera | 63 | 148 | 1 | ... | ... | 135 |
| Inflammation | ... | ... | 10 | 31 | 101 | 307 |
| Pleurisy | 14 | 6 | 10 | 5 | 4 | 39 |
| Asthma and phthisic | ... | ... | 112 | 85 | 89 | 136 |
| Consumption | 1,021 | 1,255 | 905 | 1,121 | 716 | 567 |
| King's evil, scrofula | 14 | 19 | 5 | 5 | ... | 3 |
| Dropsy | 146 | 349 | 218 | 225 | 131 | 133 |
| Apoplexy and suddenly | 47 | 30 | 48 | 55 | 49 | 59 |
| Palsy and lethargy | 14 | 17 | 12 | 18 | 19 | 28 |
| Old age, bedridden | 370 | 388 | 415 | 324 | 241 | 357 |
| Casualties | 65 | 76 | 85 | 70 | 40 | 57 |
| Childbed and miscarriages | 80 | 100 | 43 | 47 | 32 | 43 |
| Unknown causes | ... | ... | ... | ... | ... | 88 |
| Other diseases | 253 | 565 | 211 | 144 | 146 | 289 |
| Deaths in 100,000 living | 5,000 | 8,000 | 5,200 | 5,000 | 2,920 | 3,200 |

What class of diseases does it exclude? In the first place, the eruptive fevers. Among 4,095 deaths in the Equitable Society, only 1 was of smallpox. The deaths from consumption, instead of making 20, only amounted to 8·3 per cent. of the deaths, or 10·3 if those who died of ruptured bloodvessel be added. But by way of compensation, the deaths from dropsy are 22; from apoplexy and palsy 37 per 1000; instead of 11, 12, and 11, 10·6, the proportions dying of these two classes of disease in Carlisle and Philadelphia. The advantage to the assurance offices of applying tables founded on a city population in the last century, and *including all the sick*, to a class of persons in good health, and little liable to the eruptive fevers or consumption, is obvious. With a little study, the selection of long-lived persons may be carried to a still greater extent." 600.

2. Life, divided into five vicennial periods, is most secure in the second; the fatality of nearly all diseases increases afterwards in a geometrical progression. Even consumption is not an exception to this law.

3. With the civilization of a people, apoplexy increases. This is apparently not difficult of explanation. Apoplexy is, in the great majority of instances, the consequence of diseased cerebral arteries, and the latter usually coexist with hypertrophy of the left ventricle of the heart. In civilized life, the exertions of the brain, and the influences of the passions on the heart, multiply to an almost incalculable extent. What wonder that both should suffer in their organization?

"One of the most important results presented in this paper is, that the character of diseases changes in a determined ratio at different periods of existence. The tables indicate not only the degree, but the kind of danger, we have to encounter at all ages: for example, in the second vicennial period (20—40), the deaths from consumption at Carlisle constituted 50, at Philadelphia 34, in the Equitable Society 26 per cent. of the deaths from all causes; in the third vicennium the nature of the danger has altered, for the deaths from consumption contribute but 23, 28, or 11 per cent. to the entire mortality. From tables of this description, the probability of death from each class of diseases can be calculated at all ages. We will add one instance of their practical application. In the first period of life (0—20) the eruptive fevers, inflammations, scrofulous and dropsical effusions, are most to be dreaded; in Philadelphia two-fifths of the deaths were from affections of the brain and bowels. Who, with these facts before him, can fail to see the impropriety of giving children preparations of laudanum, spirits, or any food at first but the mother's bland milk? Cold often produces inflammation of the lungs in winter: but too much tenderness in this respect, and the accustoming of boys to a delicate diet, weaken the constitution. Between 20 and 40, *consumption*, inflammations, fevers, and epidemics, are the most deadly shafts of death, which, as Dr. Clark has shown, a judicious course of hygiene in this period may do much to disarm. The same class of diseases maintains the preponderance till 60; but, in the period following (60—80) *dropsies* and inflammations increase, while apoplexy gains a great ascendency. After 65 a man should undertake nothing requiring great intellectual exertion, or sustained energy: warmth, temperance, tranquillity, may prolong his years to the close of a century; a rude breath of the atmosphere, a violent struggle, or a shock, will suffice to terminate his existence. The apoplexy of the aged can, with care, be averted for several years; but it is, perhaps, the natural death, the euthanasia of the intellectual: their blood remains pure, the solids firm to the last, when a fragile artery gives way within the head,—the blood escapes, and by a gentle pressure dissolves sensibility at its source for ever." 600.

We have reached the termination of the chapter. It concludes with a few and but a few "conclusions," which wear, after the long array of figures and of facts, a rather lame appearance. They may be readily summed up. *That* governments should ensure an accurate registration—*that* all classes are ignorant of physiology, and that therefore the works and inventions of Rumford and of Arnott will be useful—*that* prevention is better than cure, and quite as easy—*that* the medical institutions of the country should be liberal—*that* "medical writers should renounce the notion that a science can be founded on the limited experience of an individual"—*that* "practical medicine cannot be taught by books, while the science of medicine cannot be acquired in the sick room—*that* morbid anatomy is indispensable—and, finally, *that*, the Government, municipal and general, should do their utmost to render towns healthy—are the inferences deduced from the statistical data set forth—inferences, most of them just, but oddly sorted, and some of them not very obviously inferential. The reader in fact is almost tempted to apply to them the pasquinade upon the ill-placed columns.—

> Care colonne che fate quà?
> Non sapiamo, in verita.
> Ah, my dear columns, what do you there?
> Of that, we've not an idea, we declare.

The author, in urging the prevention of disease, anticipates and endeavours to parry a professional objection. That objection is obvious and natural. If diseases can be materially prevented, if public hygiene proves as successful as we are told it may, what is to become of the present myriads of doctors? Mr. Farr supposes that a modified system of medical education, and of remuneration for medical services, will correct *that* evil, and will fill the gaps in the ledger and the pocket. A sanguine but extraordinary notion. The doctors, like other folks, are paid for work *done*, and it requires but a small share of economical knowledge to foretel, that their payments will decrease when there is little work to do. Fortunately even the statisticians cannot yet prevent epidemics, and once every four or five years at least, the faculty may expect to obtain a good dinner. An iron age is evidently approaching, and we recommend our brethren to seize the present moment, (the influenza is on its wing,) ere it is for ever flown. Pills will soon be a drug, draughts will be dishonoured, the only bills sent out will be bills of mortality, and they will be small and payable long after date. The statisticians themselves will, after a short season, find their occupation gone, and the dead will be too few to afford them a living. That practical philosopher, Mr. Reeve, has anticipated the coming state of the profession, and we must shortly admit with him, that "there is nothing stirring but stagnation."

The article on medical statistics, contained in the Dublin Review, and contributed by Mr. Palmer, is a clear, judicious, and well-written exposition of the causes that have contributed to the extension of human life. Addressed to the public, it is calculated to engage its attention, and to set before it in a plain yet striking manner, the means of securing health and attaining longevity. The length to which this article has run, in pursuing the more elaborate details of the preceding author, precludes the possibility of our going far with Mr. Palmer. We can only select a few points from his paper, which either illustrate those that we have already dwelt on, or supply deficiencies which *they* exhibit.

Mr. Palmer traces with care and satisfaction the causes of the demonstrative augmented value of life—a value higher in this calumniated country and climate than in any other in the world. Those causes are general and medical. Among the former, cultivation of the land and surface drainage—better clothing and better food than formerly—increased commercial prosperity, are the principal, though not the only ones. Those who talk of the "good old times," are probably ignorant of what the real characters of those times were. We would advise them to turn to our English history, and contemplate those ancestors, whose situation they conceive to have been so incomparably superior to their own. We may mention a fact or two for the benefit of those who admire so much not only the wisdom but the manners of our ancestors.

It was not till the end of the reign of Henry VIII. that any salads, carrots, turnips, or other edible roots were produced in England. Queen Catharine, when she wanted a salad, was obliged to dispatch a messenger to Flanders on purpose. In Edward the Sixth's reign, which was after Henry's,* Erasmus ascribes the frequent plagues to the nastiness, and dirt, and slovenly habits of the English. "The floors," says he, "are commonly of clay, strewed with rushes, under which lies unmolested an ancient collection of beer, grease, fragments, bones, spittle, excrements of dogs and cats, and every thing that is nasty." Hollingshed gives a lively account of the mode of living when Elizabeth came to the crown, at the close of the sixteenth century. There was scarcely a chimney to the houses, even in the considerable towns, the fire being kindled by the wall, and the smoke finding its way out as it best might. The houses were only watling, plaistered over with clay. The people slept on straw pallets, and had a good round log under their heads for a pillow. Can we wonder at plagues and epidemic fevers prevailing under such circumstances as these; or, when we recollect the filthy woollen under garments, that our ancestors, both the little and the "great unwashed," were under the necessity of wearing, can we be astonished that the present inhabitants of England should enjoy better health, and a longer range of existence, than they did?

To the medical profession itself it would be absurd to dwell on the influence which medical improvement must, *ex necessitate*, have exerted, in effecting the diminution of mortality. Yet there are one or two statistical facts which we may quote. Out of 37 cases of fever treated by Hippocrates, 21, or more than one half, died; whereas, at the Fever Hospital of London, in 1825, the total mortality was less than 1 in 7; and at the Dublin Fever Hospital, from 1804 to 1812, did not exceed 1 in 12. Independently too of the decrease of the number of cases of small-pox, the diminution of fatality in the cases that do occur is gratifying. From 1794 to 1798, the mortality, at the Small-pox Hospital of London, was 32 in 100, or nearly 1 in 3; but had diminished, in 1834, to 13 in 100, or nearly 1 in 8, a diminution which must principally be ascribed to the improved medical treatment to which the patients are subjected.

But we must pause. This article has already attained a length which precludes our adverting to further facts. As opportunities occur, we shall return to the subject of vital statistics, and endeavour to disseminate the accurate data and important conclusions that it furnishes.

---

* Anno Domini, 1553.

